# Spatio-temporal distribution of hospitalizations for chronic Chagas disease and risk factors associated with in-hospital mortality and surgical intervention in Chile

**DOI:** 10.1371/journal.pntd.0012124

**Published:** 2024-04-25

**Authors:** Nicolhole Atero, Marisa Torres, Angélica Domínguez, Benjamín Diethelm-Varela, Francisca Córdova-Bührle, Fernando O. Mardones

**Affiliations:** 1 Escuela de Medicina Veterinaria, Facultad de Agronomía e Ingeniería Forestal, Facultad de Ciencias Biológicas y Facultad de Medicina, Pontificia Universidad Católica de Chile, Santiago, Chile; 2 Departamento de Salud Pública, Escuela de Medicina, Pontificia Universidad Católica de Chile, Santiago, Chile; 3 Center for Cancer Prevention and Control (CECAN), Escuela de Medicina, Pontificia Universidad Católica de Chile, Santiago, Chile; 4 Departamento de Genética Molecular y Microbiología, Facultad de Ciencias Biológicas, Pontificia Universidad Católica de Chile, Santiago, Chile; 5 Dirección de Transferencia y Desarrollo, Vicerrectoría de Investigación, Pontificia Universidad Católica de Chile, Santiago, Chile; 6 Departamento de Enfermedades Infecciosas e Inmunología Pediátrica, Escuela de Medicina, Pontificia Universidad Católica de Chile, Santiago, Chile; Wadsworth Center, UNITED STATES

## Abstract

Chagas disease (CD) is a neglected parasitic zoonotic disease that affects over 6 million people worldwide. We conducted a retrospective study to analyze the spatiotemporal trends and risk factors for hospitalization rates of CD with cardiac and digestive diagnoses in Chile. We used the Mann-Kendall analysis for temporal trends, Global Moran’s Index, and Local Indicators of Spatial Association to identify spatial autocorrelation, and regression models to determine the risk factors associated with in-hospital mortality and surgical intervention. Between 2010 and 2020, a total of 654 hospitalizations were reported, corresponding to 527 individuals. The hospitalization rate steadily decreased over the years (t = -0.636; p = 0.009). The Global Moran’s I for the study period showed a positive spatial autocorrelation for hospitalization municipality and for residence municipality of CD patients (I = 0.25, p<0.001 and I = 0.45, p<0.001 respectively), indicating a clustering of hospitalizations in northern municipalities. The most frequent diagnosis was a chronic CD with digestive system involvement (55.8%) followed by a chronic CD with heart involvement (44.2%). The highest percentage of hospital discharges was observed among males (56.9%) and in the 60–79 age group (52.7%). In-hospital mortality risk was higher with increasing age (OR = 1.04), and in patients with cardiac involvement (OR = 2.3), whereas factors associated with the risk of undergoing a surgical intervention were sex (OR = 1.6) and diagnosis of CD with digestive involvement (OR = 4.4). The findings of this study indicate that CD is still a significant public health burden in Chile. Efforts should focus on improving access to timely diagnoses and treatment, reducing disease progression and hospitalization burden, and supporting clinicians in preventing complications and deaths.

## Author summary

Chagas disease (CD), caused by the parasite *Trypanosoma cruzi*, is a neglected tropical disease affecting 21 Latin American countries. Transmission occurs through the bite of infected bugs, blood and organ transfusions, mother-to-child transmission, and contaminated food. The disease progresses through acute there most patients are asymptomatic, or a chronic phase where around 60–70% show no symptoms for 10–30 years or life, but some may progress to a serious cardiac or digestive form. In this study, we analyze the geographical and temporal distribution of hospitalizations for chronic CD.

We utilized the national database of hospitalizations for public and private hospitals. A total of 527 hospitalized patients were observed between 2010 and 2020, corresponding to 624 hospitalizations. Our findings revealed that hospitalizations are concentrated in the northern zone and have slightly decreased over the years. The most frequent diagnosis was chronic Chagas disease (CD) with digestive system involvement (55.8%) compared to cardiac diagnosis (44.2%). However, the risk of in-hospital mortality was higher for CD with cardiac involvement, increasing with age. Meanwhile, a chronic digestive diagnosis and male sex pose a higher risk of undergoing surgery.

## Introduction

Chagas disease (CD) is a potentially life-threatening illness caused by the protozoan parasite *Trypanosoma cruzi*. It is endemic in 21 countries of Latin America, but due to an increase in human migration in recent years, a considerable number of cases have been described in Europe, Asia, and North America [1,2]. About six-eight million people worldwide are estimated to be infected, and about 12 thousand deaths are recorded annually [2,3]. *T*. *cruzi* is transmitted to humans by contact with feces/urine of infected blood-sucking triatomine bugs, and can also be transmitted through blood and organ transfusions, vertically from mother to child and orally by contaminated food or beverages [4,5].

The disease evolves from an acute to a chronic phase lasting from eight to twelve weeks and often remains undiagnosed because most patients are asymptomatic or manifest mild symptoms such as fever, malaise, and splenomegaly. In some cases, patients exhibit signs like chagoma or Romaña signs, a periorbital swelling syndrome [6]. Following infection, 60–70% of patients enter a chronic indeterminate phase for 10–30 years or even for life, characterized by positive serology, lack of symptoms, and normal electrocardiogram [7]. About 10–30% progress to a determinate form, developing mainly cardiac or digestive symptoms. The cardiac form is the most serious manifestation, developing in 20–30% of individuals, characterized by chronic cardiac failure [6,7]. The digestive form affects 15–20% of patients and is characterized by alterations in the motor, secretory, and absorptive functions of the gastrointestinal tract [6].

CD is endemic in Chile [8]. Transmission in past decades occurred mainly by the intradomiciliary nocturnal vector, *Triatoma infestans*, a triatomine bug. This route of transmission was interrupted in Chile in 1999, and ratified in 2016 by experts from the Southern Cone Initiative and Pan American Health Organization. The reproductive wild cycle of *T*. *cruzi* is maintained by the sylvatic diurnal species *Mepraia spinolai*, *M*. *gajardoi*, and *M*. *parapatrica*. These species comprise the wild animal reservoir consisting of rodents, small marsupials, rabbits, among others [9], and species that transit between the peridomestic and wild environments such as dogs, cats, goats, and other farm animals [10,11]. Surveillance encompasses mandatory notification, vector control, screening of blood and organs, as well as testing pregnant women at the first prenatal appointment, newborns, and siblings of infected mothers [8]. These strategies have identified more than 1500 cases per year in Chile in the last decade [12]. Nevertheless, CD remains underreported and misdiagnosed probably due to limited access to opportune diagnostic tests, lack of clinical awareness, and because most cases are asymptomatic [5,13]. In addition, restricted access to healthcare, or clinical referral to tertiary healthcare facilities over long distances [14], potentially leads to preventable complications or death.

Diagnosis and severity factors of CD vary according to patient comorbidities, transmission mechanisms, or geographical regions [6,15]. Nevertheless, most CD research focuses on prevalence or mortality in a specific diagnosis and few studies have described the characteristics of hospitalizations at the national level [16–18]. To our knowledge, no previous investigations have examined the epidemiological characteristics of hospitalizations related to CD in Chile and its spatial and temporal distribution. This is relevant to the health care system of the disease because hospitalizations for CD usually occur as a result of complex and untreated infections in the late stages, leading to costly surgical interventions [16].

This study aimed to (1) determine the temporal and spatial distribution of hospitalizations for chronic CD and (2) identify factors associated with in-hospital mortality and surgical intervention in chronic CD patients during 2010–2020. Our results will serve as valuable information for guiding clinical decisions and informing stakeholders involved in shaping ongoing and future public policies and integrated surveillance.

## Methods

### Ethic statement

The Scientific Ethics Committee for Health Sciences at the Pontificia Universidad Católica de Chile reviewed and approved study protocol (Resolution Act: protocol ID 2011120007 of January 07, 2021).

### Data sources

A retrospective study was conducted nationwide from hospitalized cases diagnosed with chronic CD from 2010 to 2020 (available at https://https://deis.minsal.cl/#datosabiertos). These data include hospitalizations from public and private hospitals, and the diagnoses were coded under the International Classification of Diseases (ICD-10). Two main diagnoses were included for chronic stages, CD with heart involvement (B57.2) and CD with digestive system involvement (B57.3). Acute diagnoses (B57.0 and B57.1) were not included given the interrupted vector transmission status in Chile according to official reports, so these diagnoses are likely to be overestimated as a result of reporting errors [8]. Also, P00.2 was excluded since it corresponds to congenital CD but also includes other congenital diagnoses. No records for Z22.8 were observed, as it applies to asymptomatic patients who do not require hospitalization for complications associated with the disease.

It is important to note that there may be one or more discharges for the same patient in the database due to re-admission. Nevertheless, the data was analyzed at the patient level, and re-admission data was consolidated into a single observation. Thus, information such as sex, age, municipality/region of residence and hospitalization, and date of discharge was maintained from the first record. Given that the data used had an identification code, it was possible to estimate the total number of admissions, the length of stay (sum of days hospitalized), the number of surgeries during the period, and in-hospital mortality. Number of surgeries as a numerical variable, corresponds to the number of surgeries the patient had during the whole study period, as a categorical variable, if he/she had any intervention during the period (yes/no). Hospital mortality refers to whether the patient died (yes/no) in the last discharge recorded in the study period.

### Data analysis

Demographic and clinical variables were described using proportions for categorical variables, and mean, standard deviation or median and range for numerical variables. Differences in proportions, mean and median were assessed with the Chi-squared test (*X*^*2*^), Student’s t-test, and Wilcoxon rank test, respectively. Crude and sex- and age-adjusted annual hospitalization rates were calculated through direct standardization using the World Health Organization (WHO) World Standard population and the national population data from the National Institute of Statistics (INE; Instituto Nacional de Estadísticas) [19,20]. Using “*Kendall*” package in R [21,22], the Mann–Kendall test was conducted to analyze the trend of sex and age-adjusted hospitalization rates from 2010 to 2020, aiming to identify the increasing and/or decreasing pattern over the period.

Average rates of hospitalizations were estimated along the entire period according to the residence and hospitalization municipality. The global Moran’s Index was used to assess the presence of global spatial autocorrelation for chronic CD. Values close to zero indicate spatial randomness; values approaching +1 indicate positive spatial autocorrelation and, those approaching −1, negative spatial autocorrelation [23]. Local Indicators of Spatial Association (LISA) was used to detect municipalities with significant spatial autocorrelation. Four types of clustering were identified: 1) High-High (HH) clusters occur where a municipality with high rate was surrounded by other municipalities with high values; 2) Low-Low (LL) clusters occur where a municipality with low rate was adjacent to municipalities with low values; 3) High-Low (HL) outliers occur where a high rate municipality was surrounded by low rate municipalities, and 4) Low-high (LH) outliers occur where a low rate municipality was near high values municipalities. GeoDa v1.18 and QGIS v3.34 software was used for spatial analysis [24].

Simple and multiple logistic models were conducted to understand the risk factors for in-hospital mortality and surgical intervention. The explanatory variables considered in each model included: sex, age, re-admission, and diagnosis. A stepwise model selection procedure was used to determine the most parsimonious explanatory model. Models were ranked employing Akaike Information Criterion (AIC), with the lowest AIC value considered best fitting the data. Odds ratios and 95% confidence intervals (95% CI) were reported. All analyses were conducted using R version 4.3.2 [21]. The level of significance was set at 0.05.

## Results

A total of 527 hospitalized patients were observed between 2010 and 2020, corresponding to 624 hospitalizations (15.2% had re-admission) (Table 1). CD with digestive involvement was the most reported diagnosis (55.8%). Hospitalizations were slightly more frequent in men for both digestive and cardiac diagnoses, but this difference was not significant (*p* = 0.149). The mean age of cases for the first hospitalization was significantly higher for digestive diagnoses than CD with heart involvement (68.5 and 64.3 years, respectively). When classified by age group, the highest percentage of hospitalizations was observed in patients aged 60–79 (p = 0.015). Overall, in-hospital mortality was 9.1%, higher for CD with cardiac involvement (12.4%) in contrast to digestive diagnosis (6.5%).

**Table 1:** Characteristics of patients hospitalized for chronic Chagas Disease from 2010 to 2020.

Variable	Total	Digestive	Heart	t-test/χ^2^/W	p-value
**Number of hospitalizations**	527	55.8% (294)	44.2% (233)	*X* ^*2*^ = *7*.*06*	**0.007**
**Sex**					
Male	56.9% (300)	59.9%	53.2%	*X* ^*2*^ = 2.07	0.149
Female	43.9% (227)	40.1%	46.8%		
**Age** *(mean*, *SD)*	66.6 (±16.5)	68.5 (±14.3)	64.3 (±18.8)	*t* = 2.88	**0.004**
**Age group**					
0–19	2.3% (12)	1.02%	3.7%		
20–39	3.2% (17)	3.06%	3.4%		
40–59	22.6% (119)	18.7%	27.5%	*X*^*2*^ = 12.34	**0.015**
60–79	52.7% (278)	57.8%	46.4%		
+80	19.2% (101)	19.4%	18.9%		
**Length of stay (days)** *	7 (1–654)	9 (1–654)	5 (1–100)	*W* = 36662	**< 0.001**
*(Median*, *range)*					
**In-hospital Mortality**					
Dead	9.1% (48)	6.5%	12.4%	*X*^*2*^ = 4.92	**0.026**
Alive	90.9% (479)	93.5%	87.6%		
Surgical intervention					
Yes	34.1% (175)	48.1%	16.4%	*X*^*2*^ = 55.2	**< 0.001**
No	65.9% (338)	51.9%	83.6%		
**Surgical intervention**	0 (0–3)	0 (0–3)	0 (0–2)	*W* = 42869	**< 0.001**
*(Median*, *range)*					
**Re-admission** *(≥2 admissions)*					
Yes	15.2% (78)	20.9%	8%	*X*^*2*^ = 15.43	**< 0.001**
No	84.8% (435)	79.1%	92%		
**Re-admission** *(median*, *range)*	1 (1–6)	1 (1–6)	1 (1–3)	*W* = 36662	**< 0.001**

*Sum of all hospitalization days.

The total length of stay during the study period varied between 1 and 654 days, with a median of seven days, which was higher for CD with digestive involvement. This diagnosis also had the highest percentage of surgical intervention, 48.1% compared to 16.4% for CD with cardiac involvement (χ^2^ = 55.2; p<0.001). In addition, the most common surgical procedure for digestive diagnosis was hemicolectomy (21%) and colon descent with preservation of the sphincter (15%) whereas for the cardiac diagnosis, the most frequent procedures were pacemaker placement (47.5%) and pacemaker generator replacement (17.5%) (Table 2).

**Table 2:** Surgical procedures performed on patients hospitalized due to chronic CD.

Type of surgery	N (%)
**CD with digestive involvement**	
Hemicolectomy	31 (21)
Pull through	23 (15)
Hartmann’s operation (or similar)	14 (9)
Total abdominal colectomy	12 (8)
Transit reconstitution (post Hartmann’s operation)	11 (7)
Exploratory laparotomy	9 (6)
Anterior rectal resection	9 (6)
Bowel resection and enteroanastomosis	8 (5)
Colostomy	4 (3)
Colostomy closure	3 (2)
Colonoscopy	3 (2)
Esophagectomy	3 (2)
Fecaloma (surgical treatment)	3 (2)
All other	17 (11)
Total	150 (100)
**CD with cardiac involvement**	
Pacemaker placement	19 (47,5)
Pacemaker generator replacement	7 (17,5)
Exploratory laparotomy	4 (10)
All other	10 (25)
Total	40 (100)

**Fig 1:**
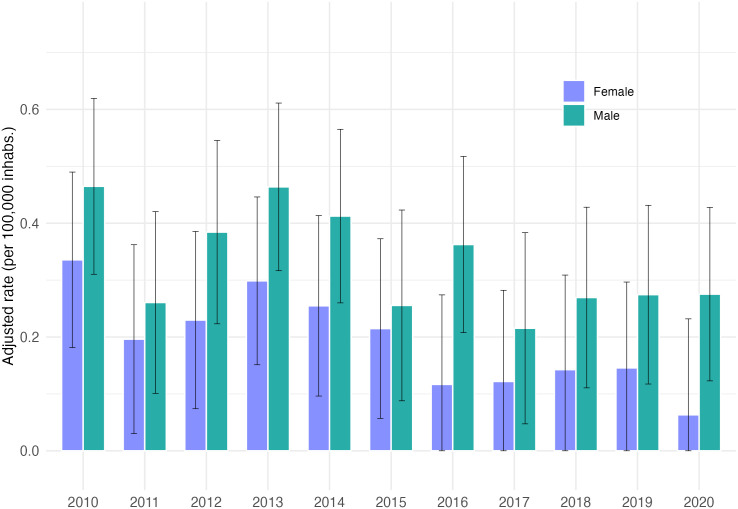
Age- and sex-adjusted hospitalization rate for chronic Chagas disease in Chile from 2010 to 2020. The error bars represent confidence intervals (CI 95%).

The annual hospitalization rate (age- and sex-adjusted) for CD over 11 years was 0.26 per 100,000 inhabitants (Fig 1 and S1 Table), and it was slightly higher for male patients (0.33 per 100,000) compared to female patients (0.19 per 100,000). The Mann-Kendall index (Table 3) showed a significant decreasing trend in overall hospitalizations over the years (t = -0.673). Although both sexes showed a decrease, it was only significant for females (t = -0.564).

**Table 3:** Mann Kendall analysis for age- and sex-adjusted hospitalization rate for chronic Chagas disease in Chile from 2010 to 2020.

Sex	tau	p-value
**Male**	-0.309	0.21291
**Female**	-0.564	0.01951
**Total**	-0.636	0.009

**Fig 2:**
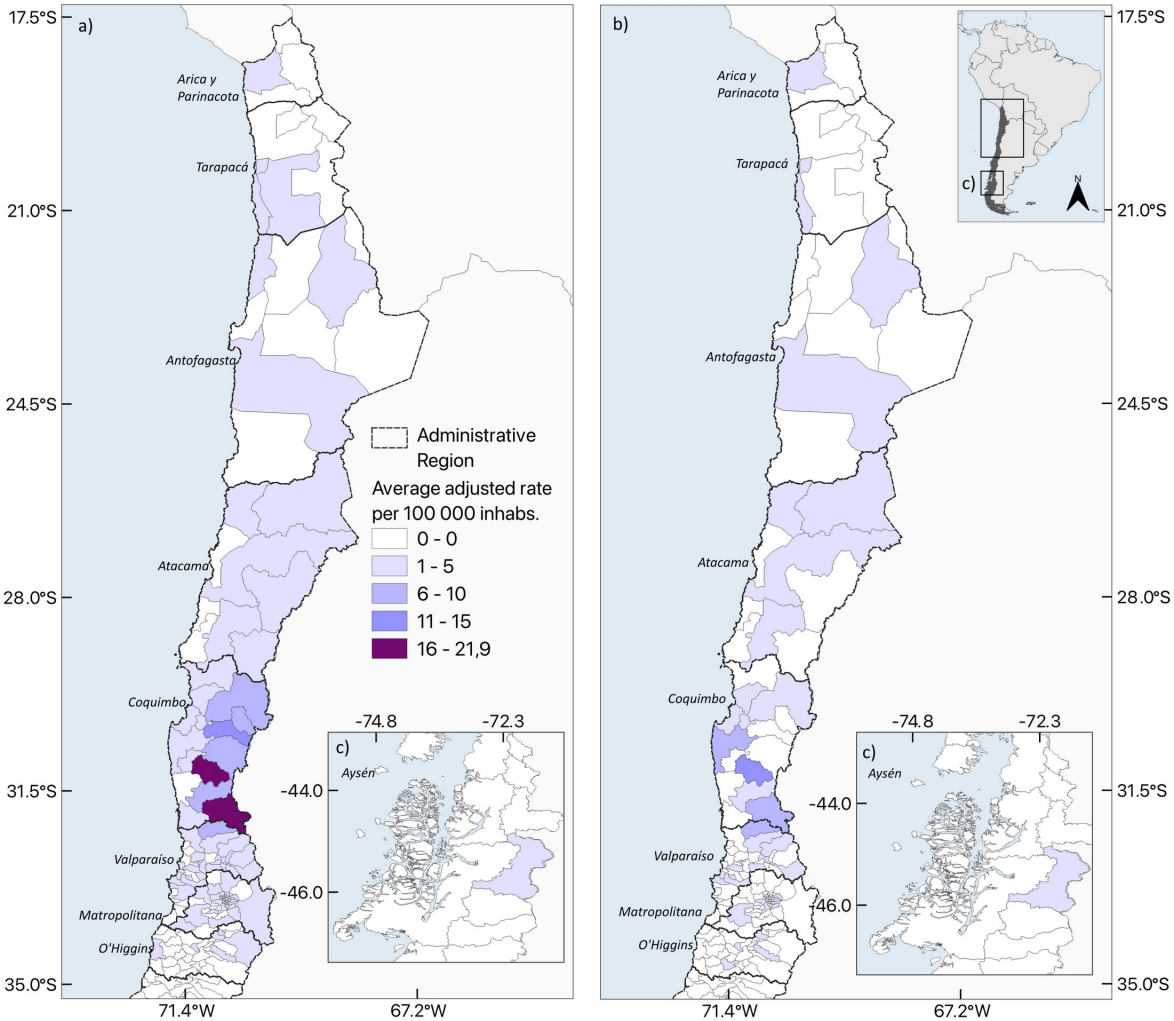
Average adjusted rate of hospitalizations of CD, according to the a) municipality of residence and b) municipality of hospitalization in Chile (2010–2019). The map was made using QGIS v3.34. The shapefile of the base layer used in this map is freely available from https://www.ine.gob.cl/herramientas/portal-de-mapas/geodatos-abiertos.

**Fig 3:**
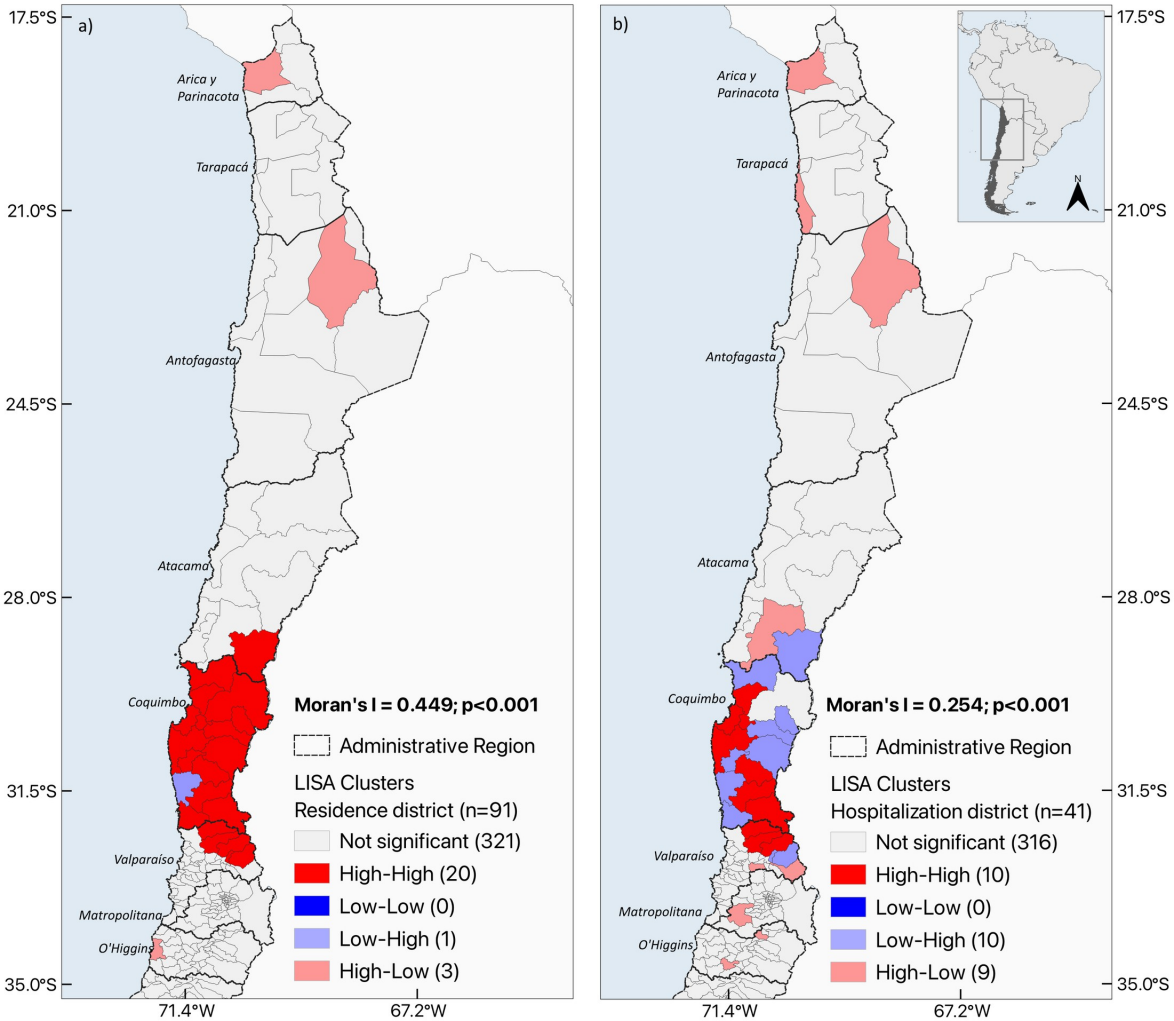
Spatial autocorrelation of the average rate of hospitalizations of CD according to the a) municipality of residence and b) municipality of hospitalization in Chile (2010–2019). The map was made using QGIS v3.34. The shapefile of the base layer used in this map is freely available from https://www.ine.gob.cl/herramientas/portal-de-mapas/geodatos-abiertos.

Hospitalizations were concentrated in the northern municipalities of the country. The average adjusted rate for the municipality of residence was 0.47 (ranging from 0 to 21.89), and the average rate for the municipality of hospitalization, it was 0.24 (ranging from 0 to 15.2). Patients hospitalized by CD resided in 26.4% (91) of the 345 Chilean municipalities (Fig 2a and S2 Table), although their hospitalizations occurred in only 12% (41) of them (Fig 2b and S2 Table). The Global Moran’s Index for the study period showed a positive spatial autocorrelation for the municipality of residence (0.449, p = 0.001) (Fig 3a). A HH cluster that included 20 municipalities located in three regions (Atacama, Coquimbo, and Valparaíso) and three HL clusters widely distributed were identified. Only one LH cluster was identified in the Coquimbo region, surrounded by municipalities with high rates. Moreover, hospitalizations showed a significant, but slightly lower spatial autocorrelation (0.254, p = 0.001) (Fig 3b). HH clusters that included 10 municipalities, located in two regions (Coquimbo and Valparaiso). This map does not show areas where autocorrelation is not significant (grey polygons), it does not imply the absence of hospitalizations in the municipality.

Results from univariate and multivariate models are presented in Tables 4 and 5. The univariate analysis indicates significant associations of in-hospital mortality for chronic CD with increased age (OR: 1.04; 95% CI: 1.02–1.1), and heart-involvement diagnosis (OR: 2.05; 95% CI: 1.1–3.8) compared to digestive diagnosis. The best multivariate models to explain in-hospital mortality included significant associations with increased age (OR: 1.04; 95% CI: 1.01–1.07) and heart-related diagnosis (OR: 2.3; 95% CI: 1.2–4.5).

**Table 4:** Univariate and multivariate analysis of the factors for in-hospital mortality of Chronic Chagas Disease.

Variable	Univariate analysis		Multivariate stepwise analysis	
	OR (95% CI)	p-value	OR (95% CI)	p-value
**Sex**				
Female	-		-	
Male	1.3 (0.7–2.4)	0.41	1.4 (0.7–2.7)	0.27
**Age (years)**	1.04 (1.02–1.1)	**0.001**	1.04 (1.01–1.07)	**<0.001**
**Number of discharges**	0.7 (0.3–1.4)	0.39		
**Number of surgical interventions**	0.5 (0.3–1.02)	0.08	0.7 (0.3–1.4)	0.38
**Diagnosis**				
Digestive	-	-	-	
Heart	2.05 (1.1–3.8)	**0.019**	2.3 (1.2–4.5)	**0.01**

AIC full model = 304.1; AIC best model = 302.1

**Table 5:** Univariate and multivariate analysis of the factors for surgical intervention for chronic Chagas Disease.

Variable	Univariate analysis		Multivariate stepwise analysis	
	OR (95% CI)	p-value	OR (95% CI)	p-value
**Sex**				
Female	-	-	-	
Male	1.7 (1.2–2.5)	**0.004**	1.6 (1.1–2.4)	**0.02**
**Age (years)**	0.99 (0.98–1.01)	0.74		
**Number of discharges**	1.8 (1.3–2.7)	**0.001**	1.4 (0.98–2.1)	0.06
**Diagnosis**				
Heart	-	-	-	
Digestive	4.7 (3.1–7.3)	**<0.001**	4.4 (2.9–6.9)	**<0.001**

AIC full model = 597.4; AIC best model = 596.7

Regarding surgical intervention, univariate models showed a significant association in male patients (OR: 1.7; 95% CI: 1.2–2.5), an increased number of discharges (OR: 1.8; 95% CI: 1.3–2.7), and digestive diagnosis (OR: 4.7; 95% CI: 3.1–7.3). In contrast, the best multivariate model indicated an increased risk of surgical intervention in males (OR = 1.6; 95% CI 1.1–2.4), digestive diagnosis (OR = 4.4; 95% CI: 2.9–6.9).

## Discussion

This study is the first that deeply analyze CD hospitalization discharges in Chile and one of the few in Latin America. CD was observed in 0.26 per 100,000 Chilean inhabitants over the study period (2010–2020), with a decreasing trend over time, and slightly higher in men than women. We found that chronic CD with digestive system involvement was the most common clinical form, although the cardiac diagnosis represents the highest risk of in-hospital mortality. Our results suggest that the highest rates of hospitalizations were observed among males, advanced age groups, and in Chilean municipalities that encompassing important CD endemic areas in the past.

The rates of hospitalizations due to CD presented here seem rather modest compared to the hospitalization rate for other infectious diseases in Chile [25–27]. Nevertheless, these results include only principal diagnoses and do not consider secondary diagnoses, which in the latest reports were 271 during the 2018–2021 period, 1.7 times higher than the 156 principal diagnoses in the same period [28]. Furthermore, it is widely recognized that CD is significantly underdiagnosed [29]. For instance, the WHO estimated that in 2010, 119,660 people were infected by *T*. *cruzi* [30], and more recent projections by Gomez-Ochoa et al. (2022) suggest an increase to 247,197 cases [2]. Nevertheless, despite these substantial estimations, around 1,500 cases are officially reported to the surveillance system annually [31]. Moreover, several cases were misclassified as acute CD and, consequently, excluded from the analysis. Finally, the interpretation of these results must consider that in the chronic phase, approximately 70% of seropositive individuals are asymptomatic (indeterminate form), whereas 30% develop serious cardiac and/or digestive pathologies [32]. Therefore, hospitalizations occur predominantly in cases of chronic diagnoses with severe symptoms that frequently require special care or surgical interventions [16].

Based on the figures mentioned above, it is crucial to highlight the financial burden of CD on the healthcare system. Ecuador has estimated US$ 300 per day hospitalized whereas Brazil reported a median cost of US$ 550 per hospitalization event, increasing to US$ 800 when surgical procedures are involved [16,17]. In Chile, the median cost per day of hospital stay is approximately US$ 400 [33,34]. Considering the total of 624 discharges, and the fact that patients diagnosed with CD usually stay in the hospital for a median of seven days, an approximate cost of US$1.7 million could be estimated for the study period, only in principal diagnoses.

The hospitalizations for chronic CD gradually decreased over the period, which may be partially attributed to improvements in the diagnosis and opportune treatment, which prevents the development of future complications, and a better understanding of the disease’s natural history. Regarding spatial distribution, there was an important autocorrelation in CD hospitalizations among the Chilean municipalities. The findings of our study revealed an important disparity between the number of municipalities where patients reside and the considerably smaller number of municipalities where hospitalization occurs. Residence municipalities are restricted to the northern zone, where high mortality has also been reported [13]. This distribution can be explained by the fact that these are municipalities with high endemicity and abundant vector transmission over the past decades [35–39]. Studies in endemic countries have reported strong spatial associations between CD cases related to environmental factors and housing characteristics [16,40,41], whereas hospitalizations are more concentrated in capitals and larger cities where complex medical care, equipment, and specialists are more likely available. This phenomenon has been consistently observed in other CD endemic countries such as Bolivia and Brazil [16,42,43]. On the other hand, individuals from socially disadvantaged backgrounds and rural origins, who already face challenges in health care access in general, encounter additional difficulties in accessing hospitals. In addition, Herazo et al. (2023) reported a 5.5 times higher of economic cost when patients were treated in a specialized reference hospital compared to when they were treated in a local primary health care hospital [14].

The spatio-temporal dynamic of CD is similar in hospitalizations and morality but differs for prevalence and notified cases. Previous studies have shown that the prevalence has remained stable over the last 65 years, with an increase in its incidence [44]. Additionally, epidemiological reports indicate that the number of notified cases has slightly increased during the last few years, except for 2020, when it decreased by 56.3% attributed to the COVID-19 pandemic [28]. As a consequence, two crucial components for the spatio-temporal distribution of CD can be identified: internal migration [45] and the heterogeneous implementation of surveillance measures in the country. The screening of blood donors began in the high endemic area in 1996 and it was extended to the entire country in 2008 [8]. Whereas screening for pregnant women started in high endemic areas in 2014 and it was extended to the entire country in 2018 [46]. In addition, the incorporation of new ICD-10 codes for asymptomatic and congenital cases in 2011, and improvements in access and coverage of healthcare services and treatment could modify these trends. All these aspects should be considered in the future of CD epidemiology and the public health system because the improvement in diagnosed cases in low endemic areas could prevent complications that require hospitalization in the future. Therefore, health personnel throughout Chile need to be trained. Also, future studies should consider the delay in CD diagnosis due to the COVID-19 pandemic, an observed phenomenon in other pathologies [47,48], since it could lead to an increase in preventable hospitalizations and deaths.

The most frequent diagnosis was CD with digestive system involvement, in contrast to chronic CD with heart involvement. This finding aligns with existing literature, which highlights that the digestive diagnoses are predominantly observed in Southern Cone countries such as Argentina, Bolivia, Paraguay, Perú, and Brazil [6,49]. However, it is important to note that the cardiac form is the most common and extensively studied diagnosis [6]. Differences in manifestations among countries have been linked to the geographical distribution of the parasite strain [50]. Sex was not associated with hospitalization diagnosis. However, previous national and international literature has shown that severity and mortality are significantly higher in men [13,40,42]. The higher prevalence and severe manifestations in men have been attributable to greater exposure due to mobility and circulation in places that favor *T*. *cruzi* transmission [42]. A significant aspect that may contribute to the observed frequency of diagnoses is that digestive CD generates a higher level of clinical suspicion. Infection with *T*. *cruzi* is globally recognized as the most common cause of acquired megacolon [51] and is the most frequent in Chile [52]. Consequently, when megacolon is detected, the probability of it being accurately reported as CD is better. On the other hand, cardiac symptoms such as arrhythmias or stroke can be attributed to numerous concurrent causes, including hypertension and atherosclerosis [15,53]. This complexity can pose challenges in diagnosis, potentially leading to underdiagnosis.

In the multivariate models, in-hospital mortality risk increases with age and in patients with cardiac involvement. The increase in mortality due to age is consistent, since the disease requires a prolonged period to develop, commonly between the third and fifth decades of life [16,17,40,54], This risk is further compounded by comorbidities such as hypertension, dyslipidemia, and ischemic conditions, which are also associated with CD in older patients [15,53]. Probably, these individuals were infected during the period of significant triatomine infestation in Chile until 1999, similar to the situation described in Brazil [16,40]. Our findings revealed a 2.6 times higher in-hospital mortality in cardiac diagnoses compared to digestive forms. This is consistent due to the cardiac diagnosis has been described as the most important clinical form of CD due to its high morbidity which leads to high work absenteeism and reduced quality of life [55,56]. Limited studies have reported in-hospital mortality. Bierrenbach et al. (2022) [16] reported an in-hospital mortality of 5.8% for the digestive form in Brazil, and Vasconez-Gonzalez et al. (2023) reported a mortality of 69.4% for severe cases[17]. However, general mortality (in and out-of-hospital) has been consistently reported to be higher in cardiac diagnosis, for instance, in Chile, Salas (2020) described the cardiac form as the most significant specific cause of CD Núñez-González et al. (2018) [41]. Additionally, reported that in Ecuador 98% of deaths were attributed to chagasic cardiopathy, compared to 2% for digestive forms, and in Brazil, Martins-Melo et al. (2021) reported 80.9% of deaths related to heart involvement compared to the 12.8% related to digestive system involvement [57].

The risk of undergoing surgery is notably higher in male patients with digestive system involvement. Specifically, the probability of undergoing surgery for digestive involvement was 4.2 times higher than for cardiac diagnoses, with 48.1% of hospitalizations involving digestive issues necessitating at least one surgical intervention, compared to 16.4% for cardiac cases. These disparities align with findings from related studies, for example, Bierrenbach et al. (2022) found that 52% of patients underwent surgical procedures for digestive diagnosis, a higher percentage than the 19.7% reported by Lima et al. (2021) for Chagas heart disease [18]. The literature suggests various surgical treatments for both diagnoses [16]. Our findings reveal that the most common surgeries in digestive diagnoses were hemicolectomy and colon descent with 21% and 15% respectively. However, more than 20 types of procedures were reported. In contrast, cardiac diagnoses involved around 10 procedures, with 47.5% involving pacemaker implantation and 17.5% requiring pacemaker battery replacements. Interestingly, despite heart transplant being described as the preferred treatment for individuals with end-stage heart failure [58], such procedures have not been performed on CD patients in Chile, as well as the use of implantable cardiac defibrillators (ICDs), used mainly in patients with malignant ventricular arrhythmias and high risk of dead [59–61].

Some strengths of our study are the use of national data and the use of CIE-10 codes, which enabled precise diagnosis identification, including the digestive form. Despite being the most common diagnosis in Chile and other countries of the region, this form has been globally neglected, with research focused on cardiac involvement [16,62]. In contrast, this study also has some limitations. Firstly, consider that Chagas disease (CD) is subject to underdiagnosis and underreporting in the surveillance system, which may extend to hospital reports. Additionally, the available data only include the principal diagnosis, excluding patients with CD as a secondary diagnosis from this study. In addition, numerous cases were erroneously categorized as acute CD. The misclassification of cases as acute CD highlights a significant error in the diagnosis process. A comprehensive understanding and in-depth knowledge of the disease are pivotal. Enhancing this understanding can lead to improved diagnosis and accurate classification of CD cases, resulting in more precise estimates and facilitating research efforts. Secondly, our use of information such as diagnosis, age, and recurrence of first discharge may introduce a bias by not accounting for changes in the characteristics of subsequent discharges.

In conclusion, our study presents a detailed analysis of the hospitalizations attributable to chronic CD in Chile, revealing that it remains a major epidemiological and public health challenge, especially in high endemic areas, and particularly for severe forms of the disease that can rapidly progress to organ complications and death. We hope our findings can help physicians recognize clinical conditions associated with higher risk for mortality and need for surgical intervention, to implement more adequate and rapid patient triaging. Efforts should focus on improving access to timely diagnoses and treatments, address the pressing need for more healthcare infrastructure and specialized professionals in high-risk regions, in order to reduce disease progression and lessen the burden of CD in hospitals.

## Supporting information

S1 Table Number and Age- and sex-adjusted hospitalization rate for chronic Chagas disease in Chile from 2010 to 2020. (DOCX)

S2 Table Average adjusted rate of hospitalizations of Chagas disease in Chile from 2010 to 2020. (DOCX)
