# Supplementary material for: Spatio-temporal distribution of hospitalizations for chronic Chagas disease and risk factors associated with in-hospital mortality and surgical intervention in Chile
Source: PLoS Negl Trop Dis. 2024 Apr 25;18(4):e0012124. doi: 10.1371/journal.pntd.0012124 (PMC11045106; doi:10.1371/journal.pntd.0012124)
Supplement: S1 Table — (DOCX) [file pntd.0012124.s001.docx]

**Table S1: Number and Age- and sex-adjusted hospitalization rate for chronic Chagas disease in Chile from 2010 to 2020.**

| **Year** | **Hospitalization (n)** | | | **Adjusted Rate** | |
| --- | --- | --- | --- | --- | --- |
|  | **Male** | **Female** | **Total** | **Male** | **Female** |
| **2010** | 40 | 36 | 76 | 0,46 | 0,34 |
| **2011** | 29 | 22 | 51 | 0,26 | 0,20 |
| **2012** | 35 | 28 | 63 | 0,38 | 0,23 |
| **2013** | 44 | 37 | 81 | 0,46 | 0,30 |
| **2014** | 40 | 29 | 69 | `0,41 | 0,25 |
| **2015** | 26 | 26 | 52 | 0,26 | 0,21 |
| **2016** | 37 | 16 | 53 | 0,36 | 0,12 |
| **2017** | 23 | 16 | 39 | 0,22 | 0,12 |
| **2018** | 30 | 17 | 47 | 0,27 | 0,14 |
| **2019** | 31 | 21 | 52 | 0,27 | 0,15 |
| **2020** | 33 | 8 | 41 | 0,28 | 0,06 |
| **Total** | 368 | 256 | 624 | 0,33 | 0,19 |
