# Supplementary material for: Spatio-temporal distribution of hospitalizations for chronic Chagas disease and risk factors associated with in-hospital mortality and surgical intervention in Chile
Source: PLoS Negl Trop Dis. 2024 Apr 25;18(4):e0012124. doi: 10.1371/journal.pntd.0012124 (PMC11045106; doi:10.1371/journal.pntd.0012124)
Supplement: S2 Table — (DOCX) [file pntd.0012124.s002.docx]

**Table S2: Average adjusted rate of hospitalizations of Chagas disease in Chile from 2010 to 2020.**

| **Region** | **Municipality** | **Hospitalization Municipality (n)** | **Hospitalization Municipality (Adj. Rate)** | **Residence Municipality (n)** | **Residence Municipality (Adj. Rate)** |
| --- | --- | --- | --- | --- | --- |
| Arica y Parinacota | Arica | 10 | 0,41 | 10 | 0,41 |
| Antofagasta | Antofagasta | 4 | 0,10 | 4 | 0,10 |
| Antofagasta | Calama | 33 | 1,80 | 35 | 1,91 |
| Tarapacá | Alto Hospicio |  |  | 3 | 0,27 |
| Tarapacá | Iquique | 15 | 0,70 | 11 | 0,51 |
| Tarapacá | Pozo Almonte |  |  | 1 | 0,57 |
| Atacama | Alto del Carmen |  |  | 1 | 1,63 |
| Atacama | Chañaral |  |  | 1 | 0,69 |
| Atacama | Copiapó | 25 | 1,41 | 22 | 1,22 |
| Atacama | Diego de Almagro | 1 | 0,62 | 2 | 1,18 |
| Atacama | Tierra Amarilla |  |  | 3 | 2,03 |
| Atacama | Vallenar | 11 | 1,82 | 10 | 1,66 |
| Coquimbo | Andacollo | 1 | 0,79 | 3 | 2,35 |
| Coquimbo | Combarbalá | 17 | 11,29 | 21 | 13,92 |
| Coquimbo | Coquimbo | 74 | 2,97 | 52 | 2,07 |
| Coquimbo | Illapel | 15 | 4,28 | 24 | 6,84 |
| Coquimbo | La Higuera |  |  | 1 | 2,22 |
| Coquimbo | La Serena | 52 | 2,20 | 27 | 1,16 |
| Coquimbo | Los Vilos |  |  | 3 | 1,27 |
| Coquimbo | Monte Patria |  |  | 11 | 3,15 |
| Coquimbo | Ovalle | 26 | 2,05 | 20 | 1,58 |
| Coquimbo | Paiguano |  |  | 4 | 8,15 |
| Coquimbo | Punitaqui |  |  | 1 | 0,77 |
| Coquimbo | Río Hurtado |  |  | 2 | 4,12 |
| Coquimbo | Salamanca | 16 | 5,40 | 36 | 12,11 |
| Coquimbo | Vicuña | 9 | 2,95 | 13 | 4,30 |
| Valparaíso | Cabildo | 3 | 1,36 | 7 | 3,17 |
| Valparaíso | Calera |  |  | 4 | 0,69 |
| Valparaíso | Calle Larga |  |  | 2 | 1,22 |
| Valparaíso | Casablanca |  |  | 1 | 0,32 |
| Valparaíso | Concón |  |  | 2 | 0,43 |
| Valparaíso | Hijuelas |  |  | 1 | 0,51 |
| Valparaíso | La Ligua |  |  | 3 | 0,74 |
| Valparaíso | Llaillay | 1 | 0,37 | 1 | 0,37 |
| Valparaíso | Los Andes | 9 | 1,24 | 6 | 0,82 |
| Valparaíso | Nogales |  |  | 1 | 0,39 |
| Valparaíso | Olmué |  |  | 1 | 0,53 |
| Valparaíso | Panquehue |  |  | 1 | 1,30 |
| Valparaíso | Petorca | 4 | 3,59 | 4 | 3,59 |
| Valparaíso | Putaendo | 3 | 1,64 | 6 | 3,25 |
| Valparaíso | Quillota | 13 | 1,29 | 3 | 0,30 |
| Valparaíso | Quilpué | 5 | 0,29 | 4 | 0,24 |
| Valparaíso | Quintero |  |  | 2 | 0,61 |
| Valparaíso | Rinconada |  |  | 3 | 2,80 |
| Valparaíso | San Esteban |  |  | 3 | 1,43 |
| Valparaíso | San Felipe | 24 | 2,82 | 15 | 1,78 |
| Valparaíso | Santa María |  |  | 2 | 1,18 |
| Valparaíso | Valparaíso | 10 | 0,30 | 9 | 0,27 |
| Valparaíso | Villa Alemana | 1 | 0,08 | 3 | 0,21 |
| Valparaíso | Viña del Mar | 20 | 0,53 | 12 | 0,32 |
| Metropolitana | Cerrillos |  |  | 1 | 0,11 |
| Metropolitana | Cerro Navia |  |  | 3 | 0,19 |
| Metropolitana | Colina |  |  | 3 | 0,18 |
| Metropolitana | Conchalí |  |  | 3 | 0,20 |
| Metropolitana | Curacaví |  |  | 1 | 0,30 |
| Metropolitana | El Bosque |  |  | 4 | 0,21 |
| Metropolitana | Estación Central |  |  | 3 | 0,17 |
| Metropolitana | Independencia | 20 | 1,93 | 5 | 0,40 |
| Metropolitana | La Florida | 4 | 0,09 | 8 | 0,19 |
| Metropolitana | Lampa |  |  | 2 | 0,18 |
| Metropolitana | Las Condes | 10 | 0,30 | 3 | 0,09 |
| Metropolitana | Lo Espejo |  |  | 1 | 0,09 |
| Metropolitana | Lo Prado |  |  | 1 | 0,09 |
| Metropolitana | Macul |  |  | 1 | 0,07 |
| Metropolitana | Maipú | 1 | 0,02 | 1 | 0,02 |
| Metropolitana | Melipilla | 3 | 0,21 | 2 | 0,14 |
| Metropolitana | Ñuñoa |  |  | 4 | 0,17 |
| Metropolitana | Paine |  |  | 1 | 0,13 |
| Metropolitana | Peñalolén | 3 | 0,11 | 6 | 0,22 |
| Metropolitana | Pirque |  |  | 1 | 0,33 |
| Metropolitana | Providencia | 5 | 0,32 | 2 | 0,12 |
| Metropolitana | Pudahuel |  |  | 2 | 0,08 |
| Metropolitana | Puente Alto | 6 | 0,09 | 4 | 0,06 |
| Metropolitana | Quilicura |  |  | 3 | 0,12 |
| Metropolitana | Quinta Normal |  |  | 4 | 0,32 |
| Metropolitana | Recoleta |  |  | 1 | 0,06 |
| Metropolitana | Renca |  |  | 1 | 0,06 |
| Metropolitana | San Bernardo | 6 | 0,18 | 3 | 0,09 |
| Metropolitana | San Joaquín |  |  | 1 | 0,09 |
| Metropolitana | San Miguel | 4 | 0,33 | 1 | 0,08 |
| Metropolitana | San Pedro |  |  | 1 | 0,84 |
| Metropolitana | San Ramón | 1 | 0,11 | 1 | 0,11 |
| Metropolitana | Santiago | 18 | 0,41 | 3 | 0,07 |
| Metropolitana | Talagante |  |  | 1 | 0,12 |
| Metropolitana | Vitacura |  |  | 1 | 0,10 |
| O'Higgins | Graneros |  |  | 1 | 0,29 |
| O'Higgins | Machalí |  |  | 2 | 0,35 |
| O'Higgins | Pichilemu |  |  | 1 | 0,54 |
| O'Higgins | Rancagua | 7 | 0,26 | 4 | 0,14 |
| O'Higgins | Rengo | 1 | 0,15 | 2 | 0,31 |
| O'Higgins | Santa Cruz | 1 | 0,23 |  |  |
| Maule | Talca |  |  | 1 | 0,04 |
